# A phenome-wide association study of ABO blood groups

**DOI:** 10.1186/s12916-020-01795-4

**Published:** 2020-11-17

**Authors:** Shun Li, C. M. Schooling

**Affiliations:** 1 School of Public Health, Li Ka Shing Faculty of Medicine, The University of Hong Kong, 7 Sassoon Rd, Pokfulam, Hong Kong, Special Administrative Region, China; 2 School of Public Health and Health Policy, The City University of New York, 55 W 125 St, New York, NY 10027 USA

**Keywords:** ABO blood group, Phenome-wide association study, Cardiovascular disease, Sex-specific analysis

## Abstract

**Background:**

ABO blood group is associated with differences in lifespan, cardiovascular disease, and some cancers, for reasons which are incompletely understood. To gain sex-specific additional insight about potential mechanisms driving these common conditions for future interventions, we characterized associations of ABO blood group antigen across the phenotype sex-specifically.

**Methods:**

We performed a phenome-wide association study (PheWAS) assessing the association of tag single nucleotide polymorphisms (SNPs) for ABO blood group antigens (O, B, A_1_, and A_2_) with 3873 phenotypes.

**Results:**

The tag SNP for the O antigen was inversely associated with diseases of the circulatory system (particularly deep vein thrombosis (DVT)), total cholesterol, low-density lipoprotein cholesterol (LDL-C), and ovarian cancer, and positively associated with erythrocyte traits, leukocyte counts, diastolic blood pressure (DBP), and healthy body composition; the tag SNP for the A_1_ antigen tended to have associations in reverse to O. Stronger associations were more apparent for men than women for DVT, DBP, leukocyte traits, and some body composition traits, whereas larger effect sizes were found for women than men for some erythrocyte and lipid traits.

**Conclusion:**

Blood group has a complex association with cardiovascular diseases and its major risk factors, including blood pressure and lipids, as well as with blood cell traits and body composition, with some differences by sex. Lower LDL-C may underlie some of the benefits of blood group O, but the complexity of associations with blood group antigen suggests overlooked drivers of common chronic diseases.

## Background

ABO blood group is associated with several diseases [1]. People with blood group O have a lower risk of cardiovascular disease (CVD) [2], including myocardial infarction (MI), peripheral vascular disease, cerebral ischemic events [3], and venous thromboembolism [4], as well as of digestive system neoplasms (gastric and pancreatic cancer) [5] and ovarian cancer [6, 7], than people with other blood groups. Reasons for these differences are not entirely clear, although lower von Willebrand factor (vWF) is thought to be one contributing factor [8]. However, the mechanisms behind these differences have not been entirely elucidated, although they could shed light on the causes of cardiovascular disease, which is increasingly realized to be incompletely understood [9, 10].

Physiologically, blood groups are manifest as many differences [11], including in red blood cell (RBC) traits, vWF, low-density lipoprotein cholesterol (LDL-C), total cholesterol, apolipoprotein E [12], and some inflammatory markers [13], such as soluble E-selectin and intercellular adhesion molecule-1. However, exactly how these differences relate to common diseases and traits has not been systematically examined, although consistent characterization may help identify informative overall patterns or overlooked causes of common conditions. ABO blood group is also associated with several diseases, such as CVD [2, 4, 14] and some cancers [7, 15, 16], whose incidence differs by sex [17, 18], highlighting the possibility of sex-specific causes and the importance of sex-specific analysis, which has rarely been conducted previously, although is increasingly realized to be relevant [19] given shorter life expectancy in men than women. To address this gap, we conducted a phenome-wide association study (PheWAS), a genotype-to-phenotype approach [12, 20] which can be performed using summary statistics [21], to examine systematically the associations of tag single nucleotide polymorphisms (SNPs) for ABO blood group antigen with a wide range of diseases and related traits, using the largest available genome-wide association studies (GWAS), with sex-specific analysis and validation where possible.

## Methods

### Blood group antigen

Four tag SNPs were used for the main ABO blood group antigens shown in Table 1 (rs8176746 for B, rs687289 for O, rs507666 for A_1_, and rs8176704 for A_2_), as previously [22]. rs507666 perfectly marks the A_1_ allele [22, 23]. Both rs8176704 [22, 24] and rs8176750 [25] (*r*^2^ = 0.99) have been previously used to mark the A_2_ allele, but the latter is limited by data availability. rs8176746 distinguishes the B allele from A [26] and was used to mark the B antigen. rs687289 is highly correlated with rs8176719 (*r*^2^ = 0.97), which determines the O allele [27, 28], but is limited by data availability. Additional file 1: Table S1 shows how the combination of alleles at these four tag SNPs for each blood group antigen corresponds to each blood group antigen by taking the unique allele [22].

**Table 1:** ABO blood group antigens and corresponding tag SNPs

Blood group antigen	Tag SNP	Effect allele/non-effect allele
A_1_	rs507666	A/G
A_2_	rs8176704	A/G
B	rs8176746	T/G
O	rs687289	G/A

### Outcomes: data sources

Large condition-specific GWAS are available from consortia for CVD, including coronary artery disease and stroke, and their risk factors (lipids, blood pressure, diabetes, and glucose metabolism), major cancers, and major contributors to the global burden of disease (including mental health, Alzheimer’s disease, and some auto-immune diseases). However, these consortia GWAS do not usually provide sex-specific summary statistics. In contrast, the UK Biobank encompasses sex-specific summary statistics for a wide range of conditions and attributes, but as a cohort study of half a million people from Great Britain intended to be aged 40 to 69 years when recruited in 2006 and 2010 [29] has a limited number of cases for rarer conditions and for diseases of old age. To be comprehensive, we largely conducted the primary analysis using the UK Biobank and then used other publicly available consortia data for the replication.

UK Biobank summary statistics were available 19,586 different phenotypes encompassing diagnoses, family history, lifestyle, current health status, anthropometrics, physical characteristics, treatment records, biochemical assays, psychological health, and physical measurements provided by Neale Lab [30]. Information concerning the source, original questionnaire or measurement, of these phenotypes is available on the official website of the UK Biobank (https://biobank.ndph.ox.ac.uk/showcase/search.cgi) keyed on the phenotype and ID (Additional file 1: Table S2). To verify previous findings of blood group on ovarian cancer [6, 7], where only self-reported ovarian cancer is available in the UK Biobank, another European ancestry-based publicly available consortium, Ovarian Cancer Association Consortium (OCAC), was included in the primary analysis.

### Outcomes: categorization

Subcategories recommended by the UK Biobank were used for inclusion and exclusion. However, the subcategories are largely reflective of the information collection approach. To make the categorization more etiologically coherent, binary outcomes were considered in groups, corresponding to selected International Classification of Diseases (ICD)-9/10 chapters, that might share causes and similar associations with blood group, i.e., circulatory, endocrine, respiratory, neoplasms, digestive, neurological, musculoskeletal, gynecologic and obstetric, hematopoietic, dermatologic, genitourinary, mental health, infectious diseases, sense organs, injuries and poisonings, symptoms, and others.

In addition to the categories above, continuous and categorical ordered phenotypes were classified in groups using the recommended categories for the UK Biobank [31], i.e., blood count, blood biochemistry, and physical measures.

### Inclusion and exclusion

**Fig. 1:**
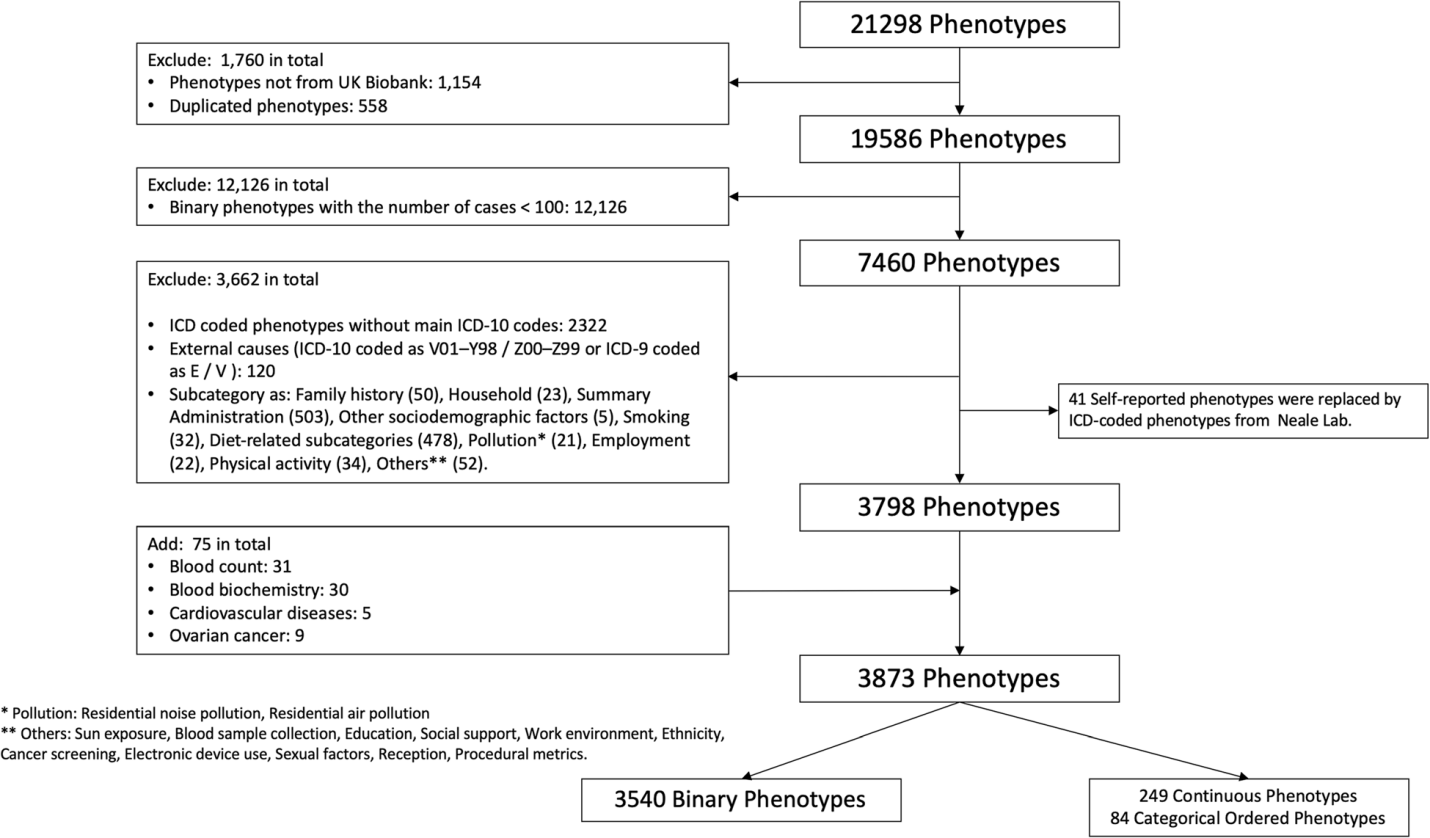
Flowchart of phenotype inclusion through the study for the 4 tag SNPs for ABO blood group antigens. ICD-10 was used to classify binary phenotypes, while continuous phenotypes were categorized with reference to the recommended UK Biobank categories and the source of information

To ensure all phenotypes examined came from the same population, non-UK-Biobank and duplicated phenotypes were excluded, unless specified otherwise. To ensure adequate power, binary phenotypes with fewer than 100 cases and continuous and categorical ordered phenotypes with the sample sizes fewer than 10,000 were excluded [32]. The ICD-coded binary phenotypes without main ICD codes or with external causes (codes as V01–Y98, Z00–Z99, or U00–U99) were also excluded. The subcategories of family history, household, summary administration, other sociodemographic factors, smoking, diet-related subcategories, pollution, employment, physical activity, and any phenotypes unrelated to health status were excluded as well. Some self-reported phenotypes were replaced by the corresponding diagnosis phenotypes in the Neale Lab consortium where available. A flowchart showing the selection of phenotypes is given in Fig. 1.

### Statistical analysis

Estimates, standard errors, effect alleles, sample sizes, and *P* values were extracted from the GWAS summary statistics using the MR-base platform [21], followed by harmonization of alleles to ensure estimates corresponded to the same effect allele for each SNP. To account for multiple comparisons, the level of statistical significance was computed as a Bonferroni correction: *P* = 0.05/Np/Ns, where Np is the number of phenotypes tested for each SNP and Ns is the number of SNPs examined [33]. As such, the significance level was 3.2 × 10^−6^ (0.05/3873/4). Differences by sex were assessed using a two-sided *z* test [34]. Estimates for binary traits obtained using linear regression were converted to odds ratios (ORs), for presentation, as necessary, using an established approximation [35].

Similar to the data extraction and harmonization process above, replication was performed using the non-UK-Biobank phenotypes excluded from the main analyses to obtain summary statistics for the same phenotypes from different consortia, i.e., the HaemGen consortium, Coronary Artery Disease Genome wide Replication and Meta-analysis plus the Coronary Artery Disease Genetics (CARDIoGRAMplusC4D), the Global Lipids Genetics Consortium (GLGC), the Nuclear Magnetic Resonance (NMR)-GWAS summary statistics, the Genetic Investigation of ANthropometric Traits (GIANT) consortium, the Meta-Analysis of Glucose and Insulin related traits Consortium (MAGIC), the UK Biobank Lung Exome Variant Evaluation (UK BiLEVE) consortium, and the BioBank Japan project. A two-sided *z* test was used to compare the primary and replication results.

## Results

**Fig. 2:**
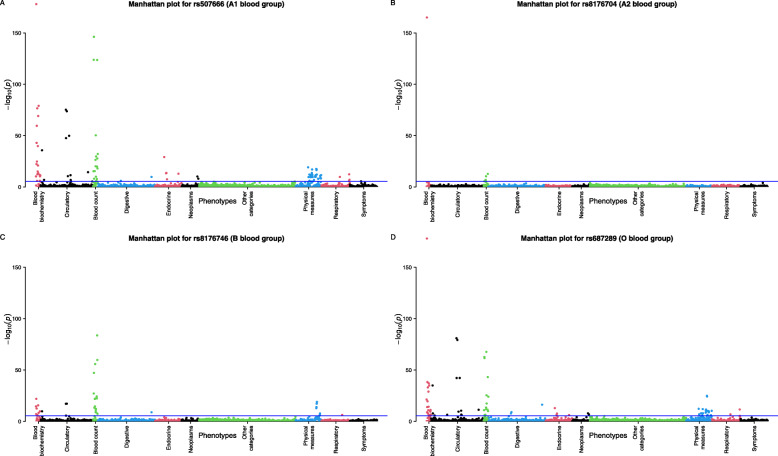
Manhattan plots for the 4 tag SNPs for ABO blood group antigens with all phenotypes included by category. The horizontal axis shows the phenotypes by category, and the vertical axis shows the -log_10_ transformed *P* values. The blue line indicates the corrected statistical significance level, *P* = 3.2 × 10^− 6^. The categories of phenotypes (from left to right on the *x*-axis) are blood biochemistry, circulatory, blood count, digestive, endocrine, neoplasms, other categories, physical measures, respiratory, and symptoms, respectively. Other categories include dermatologic, genitourinary, gynecologic and obstetric, hematopoietic, infectious diseases, injuries and poisonings, mental health, musculoskeletal, neurological, sense organs, and others. **a** Manhattan plot for rs507666 (A_1_ blood group antigen). **b** Manhattan plot for rs8176704 (A_2_ blood group antigen). **c** Manhattan plot for rs8176746 (B blood group antigen). **d** Manhattan plot for rs687289 (O blood group antigen)

In total, 3540 binary, 84 categorical ordered, and 249 continuous phenotypes were included (Additional file 1: Tables S2-S4). Of the 3540 binary phenotypes considered, 335 were circulatory, 171 endocrine, 179 respiratory, 108 neoplasms, 369 digestive, and the remaining 2378 were neurological, musculoskeletal, gynecologic and obstetric, hematopoietic, dermatologic, genitourinary, mental health, infectious diseases, sense organs, injuries and poisonings, description of symptoms, and others. Figure 2 gives Manhattan plots by tag SNP for each ABO antigen.

**Fig. 3:**
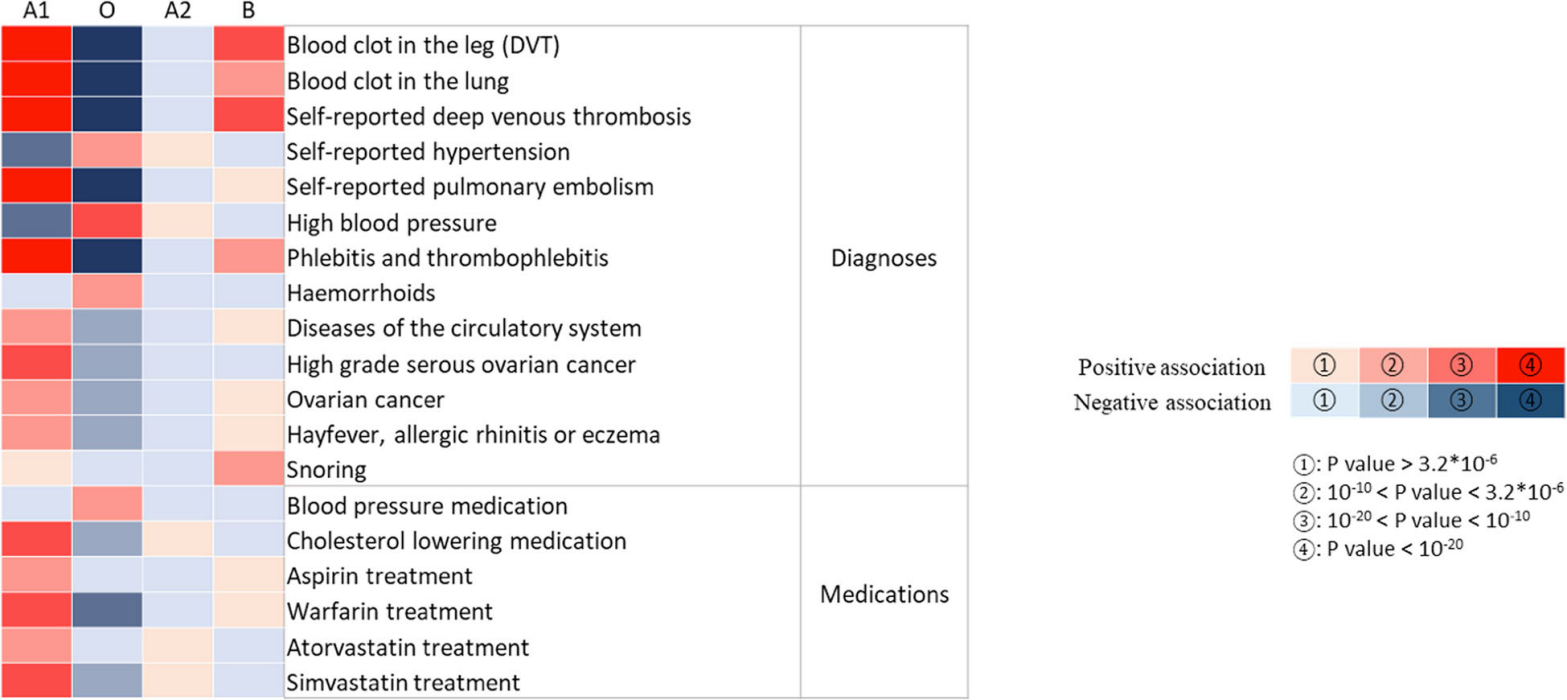
Heat maps for significant associations of tag blood group antigens with binary phenotypes. Different shades of red color represent positive associations, and different shades of blue represent negative associations. Darker colors represent smaller *P* values

For the binary phenotypes considered, ABO antigens were mainly associated with circulatory diseases (Tables 2 and 3). The direction of association differed for A_1_ and O antigens for most associations with binary phenotypes (Fig. 3), especially CVD phenotypes. The tag SNP for A_1_ antigen was positively associated with diseases of the circulatory system (OR per allele = 1.04), and with higher risks of thrombotic disorders and related treatment (blood clot in the leg, blood clot in the lung, phlebitis and thrombophlebitis, self-reported deep venous thrombosis (DVT), warfarin treatment; ORs = 1.37, 1.47, 1.64, 1.37, 1.22, respectively), but lower risk of high blood pressure (OR = 0.96). Conversely, O antigen was negatively associated with diseases of the circulatory system (OR = 0.97), and with lower risk of these thrombotic disorders and related treatments (ORs = 0.73, 0.70, 0.59, 0.73, 0.85, respectively), but higher risk of high blood pressure (OR = 1.03). Sex-specific analyses were available for high blood pressure and DVT. Directions were the same by sex with no differences by sex for high blood pressure but the effects of A_1_ and B on DVT (*P* = 0.004, 0.023, respectively) were larger in men than women.

**Table 2:** Number of significant associations of tag blood group antigens with binary phenotypes by phenotype category

Binary category	Total number	Number significant by blood group antigen			
		A_1_	A_2_	B	O
Circulatory	335	10	0	4	13
Endocrine	171	5	0	0	4
Respiratory	179	3	0	1	2
Neoplasms	108	2	0	0	2
Digestive	369	2	0	1	3
Symptoms	176	1	0	0	0
Other categories*	2202	0	0	0	0

*Other categories include neurological, musculoskeletal, gynecologic and obstetric, hematopoietic, dermatologic, genitourinary, neoplasms, mental health, infectious diseases, sense organs, injuries and poisonings, and others

**Table 3:** The number of significant associations of tag blood group antigens with continuous and categorical ordered phenotypes by category

Continuous and categorical ordered category	Total number	Number significant by blood group antigen			
		A_1_	A_2_	B	O
Blood count	31	17	6	16	14
Blood biochemistry	30	19	1	8	18
Physical measures	161	35	0	9	22
Respiratory	4	1	0	0	0
Other categories*	107	1	0	0	0

*Other categories include circulatory, endocrine, gynecologic and obstetric, neoplasms, mental health, and sense organs

O was associated with lower risk of ovarian cancer (OR = 0.93), especially for high-grade serous ovarian cancer (OR = 0.91). Conversely, A_1_ was associated with higher risk of ovarian cancer and high-grade serous ovarian cancer (ORs = 1.10, 1.14, respectively). However, no other associations with cancer were found. O was negatively associated with allergic rhinitis/eczema (OR = 0.97), while A_1_ was positively associated with this phenotype (OR = 1.04). A_1_ was also associated with early diagnosis of allergic rhinitis (effect size per allele = − 0.06). B was associated with snoring (OR = 1.06).

For treatment or medication phenotypes, A_1_ was positively associated with cholesterol lowering medication, particularly simvastatin (ORs = 1.07, 1.06, respectively), while O was negatively associated with those medications (ORs = 0.96, 0.97, respectively). Corresponding to higher risk of A_1_ antigen on thrombotic disorders, A_1_ was positively associated with aspirin (OR = 1.04). Moreover, A_1_ was associated with tonsillectomy (OR = 1.05), whereas O was negatively associated with tonsillectomy (OR = 0.96).

**Fig. 4:**
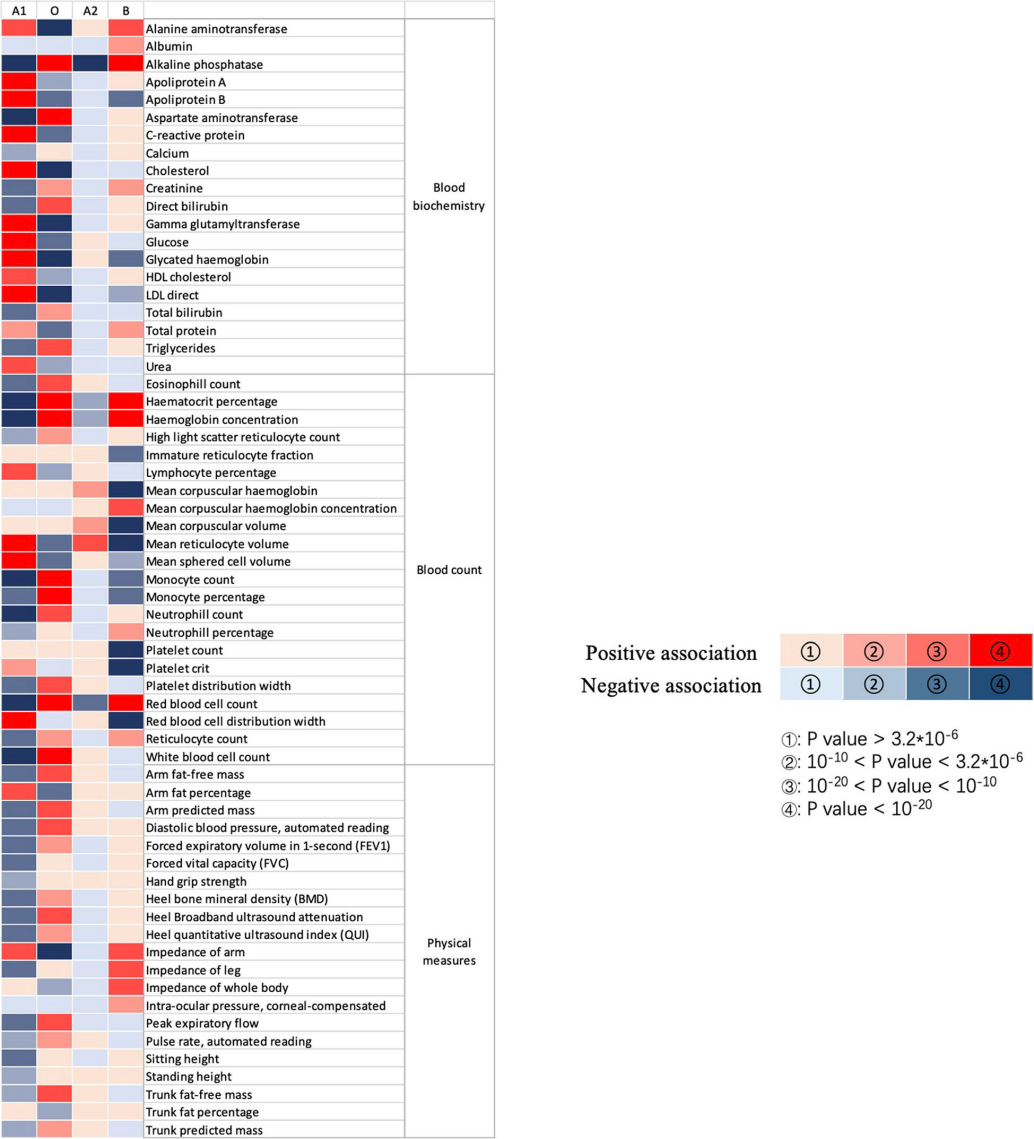
Heat maps for significant associations of tag blood group antigens with continuous phenotypes. Different shades of red color represent positive associations, and different shades of blue represent negative associations. Darker colors represent smaller *P* values

**Fig. 5:**
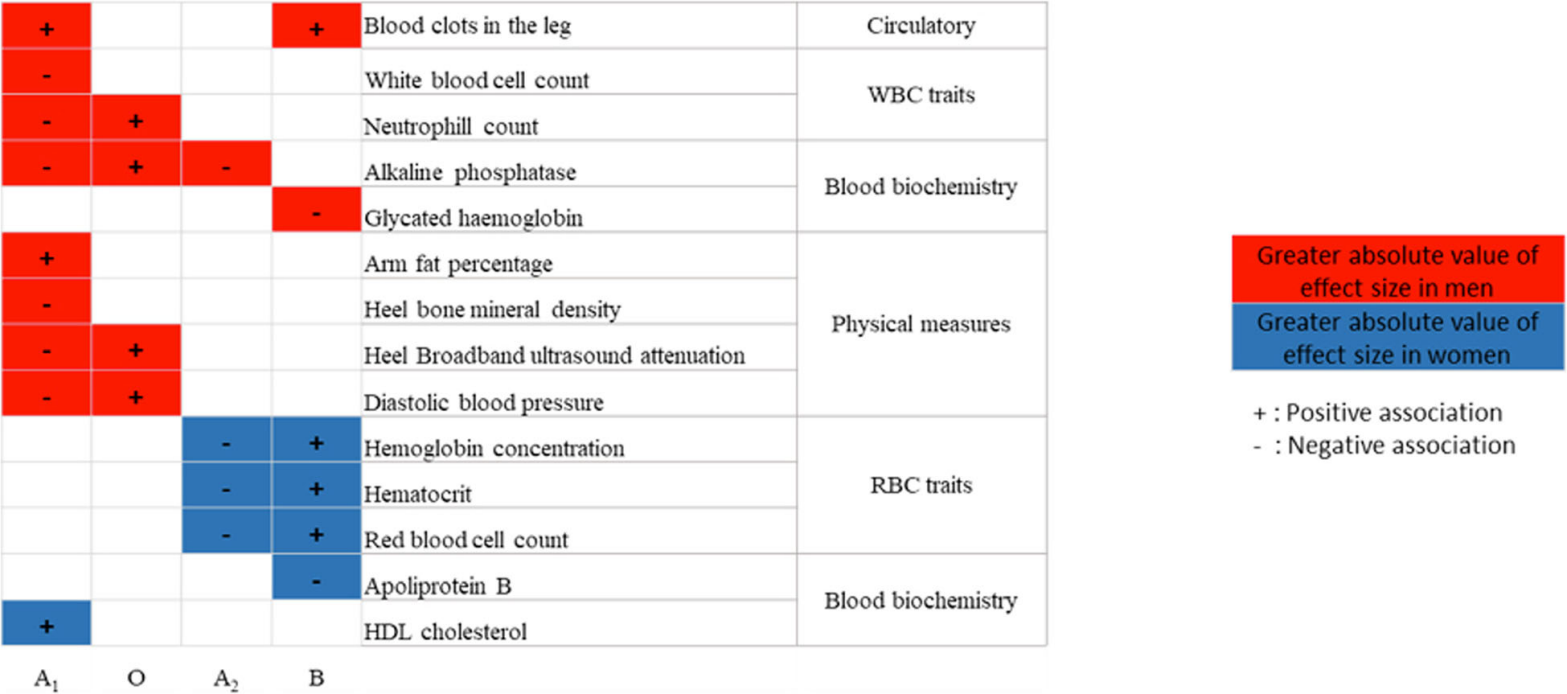
Heat map for significant sex differences in associations of tag blood group antigens with selected phenotypes. Red indicates greater absolute value of the effect size in men, whereas blue indicates greater absolute value of the effect size in women. The “+” sign represents positive associations, and the “-” sign represents negative associations

Blood group antigen had many associations with blood cell attributes and anthropometrics (Fig. 4). The tag SNP for A_1_ was associated with lower RBC traits, including hemoglobin (Hb) concentration, hematocrit (Hct) percentage, and erythrocyte count (effect sizes = − 0.063, − 0.060, − 0.062, respectively), and with lower white blood cell (WBC) traits (counts of total WBC, monocyte, neutrophil, and eosinophil, effect sizes = − 0.037, − 0.045, − 0.035, − 0.018, respectively). Differences for A_1_ in total WBC count were greater for men than women (*P* = 0.037). Similar to A_1_, A_2_ was negatively associated with RBC traits but with smaller absolute values of effect sizes (− 0.017, − 0.018, − 0.029, respectively). Conversely, B and O were associated with higher RBC traits, with B having larger effect sizes (B: 0.058, 0.044, 0.083, respectively; O: 0.034, 0.035, 0.030, respectively). B was also associated with higher mean corpuscular hemoglobin concentration (MCHC) and reticulocyte count (effect sizes = 0.039, 0.016, respectively), but lower platelet count, plateletcrit, mean corpuscular volume, mean corpuscular hemoglobin, red cell distribution width, and monocyte count (effect sizes = − 0.050, − 0.047, − 0.078, − 0.048, − 0.081, − 0.033, respectively). In contrast to A_1_, O was positively associated with those WBC traits (effect sizes = 0.026, 0.044, 0.022, 0.013, respectively). Moreover, A_2_ was positively associated with mean corpuscular volume and mean corpuscular hemoglobin (both effect sizes were 0.024). Directions of association were consistent by sex, but some differences in effect sizes were found (Fig. 5). For A_2_ and B, the associations with erythrocyte count, Hb concentration, and Hct (*P*_A2_ = 0.039, 0.020, 0.017, *P*_B_ = 0.002, 0.028, 0.013, respectively) were stronger in women than men. For A_1_ on WBC count and neutrophil count, the associations were stronger in men than women (*P* = 0.037, 0.002, respectively).

ABO blood group was also related to several aspects of blood biochemistry (Fig. 4). A_1_ was associated with higher levels of many lipoprotein traits, particularly LDL-C, high-density lipoprotein cholesterol (HDL-C), total cholesterol, apolipoprotein A (ApoA), apolipoprotein B (ApoB) (effect sizes = 0.058, 0.022, 0.058, 0.030, 0.042, respectively), glucose phenotypes, alanine aminotransferase (ALT), and C-reactive protein (effect sizes = 0.031, 0.019, 0.032, respectively), while O was negatively associated with these phenotypes (effect sizes = − 0.032, − 0.012, − 0.030, − 0.015, − 0.020, − 0.021, − 0.023, − 0.024, respectively). A_1_ was associated with lower levels of liver biochemistry, i.e., alkaline phosphatase (ALP), aspartate aminotransferase (AST), creatinine, triglycerides, direct bilirubin, and total bilirubin (effect sizes = − 0.322, − 0.048, − 0.018, − 0.020, − 0.026, − 0.021, respectively), whereas O had opposite directions of association with these phenotypes (effect sizes = 0.254, 0.032, 0.012, 0.017, 0.018, 0.016, respectively). Among the above, the absolute value of effect sizes for A_1_ (effect sizes = 0.057, 0.022, respectively) was almost double than that for O (effect sizes = − 0.032, − 0.012, respectively) with opposite directions of association with LDL-C and HDL-C. B was also related to lower ApoB and LDL-C in both sexes together (effect sizes = − 0.036, − 0.029, respectively) and in women (effect sizes = − 0.048, − 0.037, respectively), but not in men (effect sizes = − 0.023, − 0.020, respectively), with a significantly larger effect size for ApoB among women than men (*P* = 0.015). B was also associated with higher levels of ALT, ALP, albumin, and creatinine (effect sizes = 0.037, 0.048, 0.028, 0.020, respectively), and lower levels of glycated hemoglobin (HbA1c) (effect size = − 0.039). A_2_ was only negatively associated with ALP (effect size = − 0.141). Stronger associations in men than women were evident for the associations of ALP with A_2_, B, and O, and in the negative association of B with HbA1c (*P* values < 0.05), whereas the association of A_1_ with HDL-C with was stronger in women than men (*P* = 0.035).

ABO blood group was also associated with several measures related to the cardiovascular and respiratory systems, including blood pressure and spirometry. A_1_ was associated with lower diastolic blood pressure (DBP), pulse rate, forced vital capacity (FVC), and forced expiratory volume in 1 s (FEV_1_) (effect sizes = − 0.024, − 0.012, − 0.014, − 0.015, respectively), while O was positively associated with DBP, pulse rate, and FEV_1_ (effect sizes = 0.016, 0.012, 0.010, respectively). Stronger associations were found in men than women for the negative association of blood group A_1_ antigen with DBP (effect sizes = − 0.028, − 0.014, respectively), and the positive association of blood group O antigen with DBP (effect sizes = 0.022, 0.011, respectively). No significant differences by sex were found for pulse rate, FVC, or FEV_1_.

ABO blood group was also associated with physical attributes (Fig. 4), including anthropometry. A_1_ was associated with lower heel broadband ultrasound attenuation (BUA), heel quantitative ultrasound index (QUI), heel bone mineral density (BMD), and standing and sitting height (effect sizes = − 0.028, − 0.023, − 0.023, − 0.008, − 0.014, respectively), whereas O was positively associated with BUA, QUI, and BMD (effect sizes = 0.019, 0.016, 0.016, respectively). A_1_ was related to higher arm fat percentage and impedance of the arm but lower arm fat-free mass (effect sizes = 0.013, 0.016, − 0.010, respectively), while O had opposite directions of association with these three traits (effect sizes = − 0.011, − 0.016, 0.008, respectively). In addition, B was associated with higher impedance of the arm and leg, and intra-ocular pressure (effect sizes = 0.022, 0.032, 0.044, respectively). Directions of associations were generally similar by sex, although stronger associations in men than women were evident for the negative associations of A_1_ antigen with arm fat-free mass, heel BUA, and BMD (men: − 0.024, − 0.045, − 0.043; women: − 0.009, − 0.018, − 0.010; *P* values < 0.05), and in the positive associations of O antigen with heel BUA and BMD (men: 0.030, 0.028; women: 0.013, 0.008; *P* values < 0.05), where associations were not evident among women.

Replications (Additional file 1: Table S5), where available, showed consistent directions of associations as the primary analysis with most replication results having similar effect sizes to the primary ones, particularly for the negative associations of A_1_ with white blood cell traits, creatinine, and standing height; of A_2_ with Hct and Hb; of B with ApoB, HbA1c, LDL-C, and monocyte traits; and of O with LDL-C, HDL-C, glucose, urea and C-reactive protein, and in the positive associations of A_1_ with ApoB, HDL-C, and urea; of A_2_ with MCV and MCH; of B with MCHC, Hct, albumin, and creatinine; and of O with Hct, creatinine, and white blood cell traits.

## Discussion

Consistent with previous findings, O blood group antigen was associated with lower risk of circulatory diseases, particularly DVT [2], lower risk of ovarian cancer [6, 7], and higher levels of several red blood cell traits [11], whereas the direction of these associations is opposite for blood group A [2, 6, 7]. Our study adds by differentiating the effects of A_1_ and A_2_ antigens, by giving effects by sex and by subtype of some diseases, and by showing that these differences for O and A_1_ antigens extend to white blood cell traits, specific blood biochemistry, and body composition.

As in the relevant existing literature, O blood group was protective and A was harmful for CVD [2, 4, 14]. In addition, only the A_1_ subgroup rather than A_2_ was related to higher risk of CVD. We also found some indications that men had greater risk than women, for example for the association of A_1_ with DVT (*P* = 0.004).

The protective effects of blood group O and harmful effects of A_1_ antigen on ovarian cancer, particularly for high-grade serous ovarian cancer classified as type 2 ovarian cancer clinically and genetically [36], are consistent with previous findings [6, 7]. We also found that associations of O with type 1 ovarian cancers [36] (low grade or low malignant serous/mucinous ovarian cancer, or clear cell ovarian cancer, or endometrioid ovarian cancer) were not significant (*P* values > 0.05).

In other relevant literature, ABO blood group has shown inconsistent associations with hypertension and blood pressure [37–39]. Here O was also positively associated with diastolic blood pressure in the UK Biobank, consistent with a population in central Asia [40], whereas the inverse association of A with DBP has also been reported in people of African ancestry [41]. Consistent with previous findings, O was negatively associated with LDL-C [42] and A_1_ positively with LDL-C [43, 44]; however, A_2_ was not related to LDL-C. B was also related to lower LDL-C, although the magnitude was larger for O. In addition, O was also associated with lower levels of several other lipid traits, including HDL-C, total cholesterol, ApoA, and ApoB. Differences by sex were evident for LDL-C, where associations with A_1_ and B antigens were stronger in women than men. Associations in the ABO gene with total cholesterol and LDL-C are consistent with GWAS [45, 46], so some effects of blood group on CVD may be due to LDL-C [47]. Meanwhile, the direction of associations with A_1_ was opposite to O, which may be relevant to CVD [48, 49], and has some consistency with previous findings [43, 44, 50]. Blood group was not associated with type 2 diabetes, where previous findings have been inconsistent [51, 52], while we show that O and B antigens are protective for glucose metabolism. Although O was associated with higher levels of some liver biochemistry phenotypes (ALP, AST) but lower ALT, a Mendelian randomization study showed that ALP or AST are not relevant to coronary artery disease, but ALT might be a protective factor for coronary artery disease [53], which is consistent with our findings.

Despite few systematic studies of the relation of blood group with body composition, we found similar associations. Blood type O was associated with both lower LDL-c and higher bone mineral density, consistent with a Mendelian randomization study showing a negative causal association between them [54]. O was also associated with lower body fat and higher muscle mass and vital capacity, similar to the short-term effects of testosterone [55].

The inverse associations of the tag SNP for A_1_ (rs507666) with Hb concentration and Hct, and the positive associations of O antigen with these attributes are consistent with a meta-analysis in Europeans [11]. We provide new evidence that the B antigen is associated with lower platelet count and plateletcrit, and A_1_ is related to lower platelet distribution width, possibly because of ABO modulation of platelet surface or platelet function [56, 57]. Few previous studies have considered the relation of ABO blood group antigens with platelets in detail [58].

The negative association of O antigen with allergic rhinitis/eczema indicates effects of O antigen on the immune system. Opposite patterns of association for A_1_ and O were also found for several white blood cell traits, particularly counts of monocytes, granulocytes, and neutrophils. O antigen is also associated with lower risk of severe acute respiratory syndrome coronavirus (SARS-CoV), where anti-A antibodies might block the receptor-binding process of SARS-CoV spike protein on angiotensin-converting enzyme 2 [59]. A recent study about coronavirus disease 2019 (COVID-19) from China also provides evidence of lower risk in the O blood group, and greater vulnerability to infection for blood group A [60], suggesting linkage between blood group and the immune system. Besides ABO blood group influencing inflammation, it also influences coagulation, for example vWF, although exactly how these factors together mediate effects of blood group on CVD is not well understood. It has been suggested that inflammatory processes may be more important for individuals with O blood group while coagulation may be more important for non-O individuals [61]. Elucidating the relative role of inflammation and coagulation in driving the effects of blood group could also inform the development of new interventions for CVD.

Despite conducting a systematic search in the largest available studies, this study has some limitations. First, not all phenotypes of interest were available, most notably vWF which is well-known to be associated with ABO blood group. Second, insufficient cases were available to test some associations, such as with malaria and pancreatic cancer. Some phenotypes, such as *H. pylori*-related phenotypes, were classified as secondary ICD codes and therefore were not included in the study. Third, by comparing the tag SNPs for the main ABO blood group antigens, we are providing effects of ABO antigens rather than ABO blood groups. However, presenting effects of ABO antigens provides more information than blood groups [24] and is more relevant to the key questions of how and why ABO blood groups matter, so as to inform the development of interventions. Fourth, the consortium used for most phenotypes was the UK Biobank, which is not representative of the UK population. However, the criteria for internal validity are no confounding and no selection bias. Like all studies in older adults, the UK Biobank may be open to selection bias, from death prior to recruitment, which is likely to generate most bias for conditions subject to competing risk from earlier onset diseases that share etiology with the disease of interest [62]. Such bias may explain why, unlike previous observational studies [2, 3], we did not find an association of ABO blood group with stroke. Fifth, because of data availability, the underlying studies largely relate to populations of European descent. It would be immensely valuable to repeat the analysis when suitable studies more representative of the global population become available, particularly as the geographical distribution of blood groups varies [63]. The underlying studies were adjusted for population stratification, as appropriate, but given much of the information came from the UK Biobank, it is possible that some confounding due to the geographical distribution of blood groups in Great Britain remains.

## Conclusion

After systematic examination, we found that tag SNPs for ABO blood group antigens are associated with CVD, red blood cell traits, white blood cell traits, lipid metabolism, and the musculoskeletal system. Some associations for CVD and the musculoskeletal system were stronger in men, while associations with blood traits and lipid metabolism were stronger in women. Lower LDL-C may underlie some of the benefits of blood group O, but the complexity of associations with blood group suggests overlooked drivers of common chronic diseases.

## Supplementary information

**Additional file 1: Table S1.** ABO blood group antigens and corresponding tag SNPs. **Table S2.** The associations of four tag SNPs for ABO blood group antigens with 3873 phenotypes. **Table S3.** The number of binary phenotypes and characteristics of their cases by disease category. **Table S4.** The number of continuous and categorical ordered phenotypes included and characteristics of their sample sizes in each category. **Table S5.** Comparison between study results and replication results.

## Data Availability

The datasets generated and analyzed during the current study are available in the UK Biobank repository, [https://docs.google.com/spreadsheets/d/1kvPoupSzsSFBNSztMzl04xMoSC3Kcx3CrjVf4yBmESU/edit?ts=5b5f17db#gid=178908679], and MR-base repository, [https://gwas.mrcieu.ac.uk/phewas/].

## Abbreviations

ALP: Alkaline phosphatase
ALT: Alanine aminotransferase
ApoA: Apolipoprotein A
ApoB: Apolipoprotein B
AST: Aspartate aminotransferase
BMD: Bone mineral density
BUA: Broadband ultrasound attenuation
CARDIoGRAMplusC4D: Coronary Artery Disease Genome wide Replication and Meta-analysis plus The Coronary Artery Disease Genetics
COVID-19: Coronavirus disease 2019
CVD: Cardiovascular disease
DBP: Diastolic blood pressure
DVT: Deep vein thrombosis
FEV_1_: Forced expiratory volume in 1 second
FVC: Forced vital capacity
GLGC: The Global Lipids Genetics Consortium
GWAS: Genome-wide association studies
Hb: Hemoglobin
HbA1c: Glycated hemoglobin
Hct: Hematocrit
HDL-C: High-density lipoprotein cholesterol
ICD: International Classification of Diseases
LDL-C: Low-density lipoprotein cholesterol
MCHC: Mean corpuscular hemoglobin concentration
OCAC: Ovarian Cancer Association Consortium
OR: Odds ratio
PheWAS: Phenome-wide association study
QUI: Quantitative ultrasound index
RBC: Red blood cell
SARS-CoV: Severe acute respiratory syndrome coronavirus
SNP: Single nucleotide polymorphism
vWF: Von Willebrand factor
WBC: White blood cell
